# Lactylation, an emerging hallmark of metabolic reprogramming: Current progress and open challenges

**DOI:** 10.3389/fcell.2022.972020

**Published:** 2022-08-26

**Authors:** Xuelian Liu, Yu Zhang, Wei Li, Xin Zhou

**Affiliations:** ^1^ Cancer Center, The First Hospital of Jilin University, Changchun, China; ^2^ Department of Clinical Laboratory, The Second Hospital of Jilin University, Changchun, China; ^3^ Cancer Research Institute of Jilin University, The First Hospital of Jilin University, Changchun, China; ^4^ International Center of Future Science, Jilin University, Changchun, China

**Keywords:** lactate, lactylation, posttranslational modification, epigenetic, metabolism

## Abstract

Lactate, the end product of glycolysis, efficiently functions as the carbon source, signaling molecules and immune regulators. Lactylation, being regulated by lactate, has recently been confirmed as a novel contributor to epigenetic landscape, not only opening a new era for in-depth exploration of lactate metabolism but also offering key breakpoints for further functional and mechanistic research. Several studies have identified the pivotal role of protein lactylation in cell fate determination, embryonic development, inflammation, cancer, and neuropsychiatric disorders. This review summarized recent advances with respect to the discovery, the derivation, the cross-species landscape, and the diverse functions of lactylation. Further, we thoroughly discussed the discrepancies and limitations in available studies, providing optimal perspectives for future research.

## 1 Introduction

Metabolic reprogramming describes how metabolism plays a role in regulating numerous pathophysiological settings, which is a rapidly growing area of research (Xie et al., 2016; Wang et al., 2018; Faubert et al., 2020; Sun L. et al., 2021). During this process, many metabolites can serve as substrates of posttranslational modifications (PTMs), leading to epigenetic changes, which play critical roles in heritable, reversible and fine regulation of cellular plasticity (Flavahan et al., 2017; Thakur and Chen, 2019). For example, classic metabolites, including acetyl coenzyme A (acetyl-CoA) and S-adenosylmethionine, can be used by acetyltransferases for lysine acetylation and by methyltransferases for lysine methylation, respectively (Dancy and Cole, 2015; Luo, 2018). Lactate, originally thought to be a metabolic waste product of glycolysis, has recently been reconceptualized to play a pleiotropic role in shaping cell identities through metabolic rewiring and epigenetic modifications (Ferguson et al., 2018; Magistretti and Allaman, 2018; Ippolito et al., 2019; Brooks, 2020; Rabinowitz and Enerbäck, 2020; Brooks et al., 2022). In 2019, Zhang et al. found for the first time that lactate can drive histone lactylation and modulate gene transcription directly, where it exerts the non-metabolic functions (Zhang et al., 2019). Based on their observations, a mounting number of studies focused on protein lactylation sharply emerged over the next 3 years. Here, we provide a broad overview of lactate-derived lactylation, a new contributor to the epigenetic landscape, from biogenesis to functions in physiological and pathological processes, that is, what we know and what we achieve. We also further summarize and discuss gaps between different studies to provide optimized directions for future research.

## 2 Source, metabolism and shuttle of lactate

Lactate, a hydroxy carboxylic acid produced during glycolysis, includes two stereoisomers, termed as the left-handed (l-lactate) and the right-handed (d-lactate) (Manosalva et al., 2021). Compared to nanomolar level for d-lactate, l-lactate is the predominant physiological enantiomer, with a physiological serum concentration of 1–2 mM(Levitt and Levitt, 2020).

In glucose metabolism, l-lactate dehydrogenase (LDH) catalyzes the bidirectional conversion of pyruvate and l-lactate in both hypoxic and normoxic conditions (i.e., the Warburg effect). An increase level of L-LDHA and l-lactate has been confirmed in neoplastic (Xiang et al., 2018; Liu et al., 2021) and inflammatory disease (Song et al., 2019; Souto-Carneiro et al., 2020; Zhang M. et al., 2021; Kvacskay et al., 2021). In contrast, D-LDH only mediates the one-way conversion from d-lactate to pyruvate (Levitt and Levitt, 2020). d-lactate is derived from the methylglyoxal (MGO) pathway, presenting the process from S-lactoylglutathione (LGSH) to d-lactate by glyoxalase 2 (GLO2)-induced catalysis (Allaman et al., 2015). Several lines of evidence observed elevated level of d-lactate under pathophysiological conditions, including short-bowel syndrome (Oh et al., 1979; Zhang et al., 2003), fatigue syndrome (Sheedy et al., 2009), diabetes (Kondoh et al., 1992), propylene glycol intoxication (Jorens et al., 2004), d-lactate encephalopathy (Gibbs and Hertz, 2008; Ling et al., 2012) and also patients harboring deleterious enzymatic variants (i.e., D-LDH mutations) (Monroe et al., 2019).

Lactate cross the cell membrane by three known ways: free diffusion, the anion-exchange and transporters. Specific transporters contribute the most in the lactate shuttle. These shuttles, primarily including monocarboxylic acid transporters (MCTs), are modulated by the concentration gradient, the pH gradient and the redox state (Merezhinskaya and Fishbein, 2009). MCTs, mainly MCT1 and MCT4, are present ubiquitously, conferring free access to lactate for almost all cells. MCT1 is associated with lactate import, while MCT4 is referred to lactate export. High expression of MCT4 is typically observed in highly glycolytic cells and is commonly in response to hypoxic conditions, which meet the growing need of lactate export (Luo et al., 2017). In addition, the lactate shuttles mediate the process of the reverse Warburg effect. A classic example is the astrocyte-neuron lactate shuttle hypothesis, holding that neurons can metabolize lactate produced by astrocytes and consecutively released glutamate, in turn, for astrocytes uptake (Pellerin and Magistretti, 1994). Similarly, lactate produced in muscle can be re-utilized by gluconeogenesis in liver, eventually incorporating into the Cori cycle (Rubin, 2021).

Collectively, the lactate source, metabolism and shuttles provide evidence on various roles of lactate in pathophysiological conditions. Lactate has been identified as a biomarker in many diseases, such as neoplastic (Ippolito et al., 2019), inflammatory and autoimmune diseases (Jansen et al., 2009; Pucino et al., 2017). To date, accumulating evidence reveals that the “updated-lactate” can serve as an energy source (Bergman et al., 1999; Jin et al., 2019), a signaling molecule (Brooks, 2009; Tang et al., 2014) and an immunoregulatory molecule (Colegio et al., 2014; Zhou et al., 2021). That is, lactate plays important roles in regulating metabolic pathways, tumor angiogenesis, immune response and cell-to-cell communication within the tumor microenvironment. Nevertheless, the mechanisms by which lactate regulates these metabolic and cellular functions remain unclear. Lactylation provides a resource that can act as a starting point for deeper dissection of lactate metabolism, identification of physiological and pathological mechanisms and eventual translation of relevant discoveries into clinical practice.

## 3 Lactylation: A novel posttranslational modification

### 3.1 Discovery of lactylation

**FIGURE 1:**
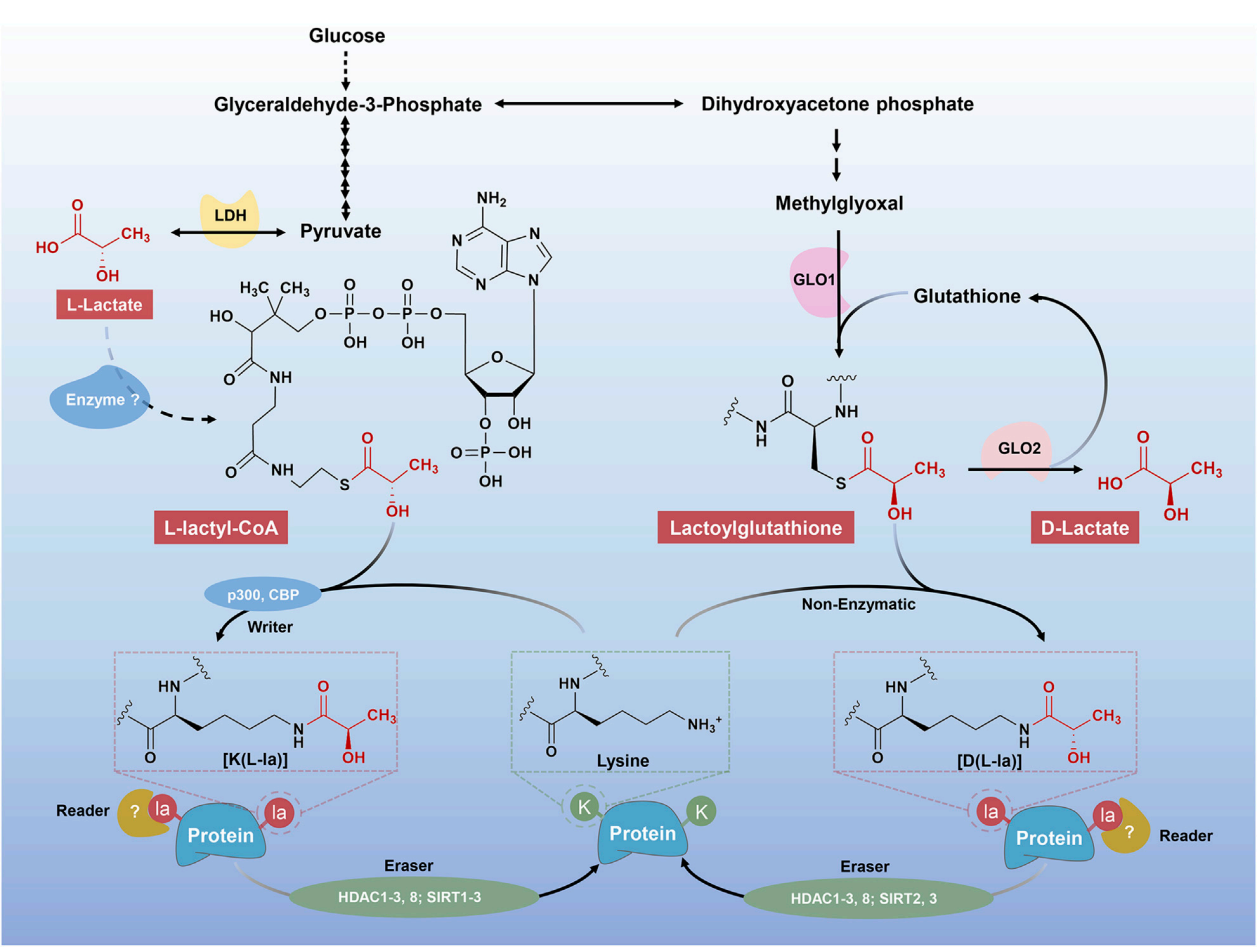
Different mechanisms of “lactylgenesis”. Two forms of Kla have been described, including K (L-la) and K (D-la). The K (L-la) proposed by Zhang et al. described an enzymatic mechanism, where l-lactate was first transformed into its activated form, L-lactyl-CoA. L-lactyl-CoA then acts as a direct substrate for K (L-la), transferring lactyl groups to lysine residues on histones as the aid of “writers”, including p300 and CBP. The K (D-la) presents a non-enzymatic process from MGO, LGSH to lactoyllysine as described by Gaffney et al. The K (D-la) would presumably accumulate in the absence of a dedicated “eraser” protein, meaning that it could be tightly regulated by the “erasers”, such as HDAC1, 2, 3, 8 and SIRT2, 3.

Metabolic activities are fundamental to living systems. Metabolic end products and intermediates not only serve as their cognate roles but also signaling molecules, where they exert non-metabolic functions. A classic example is the acetylation of histone lysine residues (Kac), which is derived from acetyl-CoA, a cellular metabolite produced in the Krebs cycle (Chen et al., 2018; Wang et al., 2022). Inspired by the discovery of Kac, lysine lactylation (alternatively named as lysine lactoylation, Kla) was recently identified by two independent research teams (Zhang et al., 2019; Gaffney et al., 2020). According to them, two forms of Kla, termed L-lactyllys [K (L-la)] and D-lactoyllys [K (D-la)], have been described (Figure 1).

In 2019, Zhang et al. firstly proposed histone K (L-la), a new type of PTM, which can be labeled by l-lactate (Zhang et al., 2019). Being detected by the high-performance liquid chromatography (HPLC)-tandem mass spectrometry (MS/MS) analysis, a mass shift of 72.021 Da on lysine residues of tryptically digested core histones from human MCF-7 cells was observed, which was subsequently confirmed to be the l-lactate-derived histone K (L-la) with the aid of isotope sodium l-lactate (^13^C3) (Zhang et al., 2019). Later, Gaffney and others suggested that the modification, K (D-la), was widely present in cells via MS(Gaffney et al., 2020). Of note, there are some differences between the two forms of Kla, including the source and the chirality of substrates, the target proteins and the functional phenotypes (Figure 1).

Firstly, Zhang et al. perceived that L-lactyl-CoA, the activated form of l-lactate, is the substrate for histone K (L-la) under actively enzymatic conditions (Zhang et al., 2019). In contrast to the view of Zhang et al., however, Gaffney and others proposed a passively non-enzymatic condition, where LGSH was transferred to protein lysine residues, generating a K (D-la) modification on proteins (Gaffney et al., 2020). They also demonstrated that GLO2 played an important role in modulating LGSH and K (D-la) (Gaffney et al., 2020).

Secondly, Zhang et al. highlight the K (L-la) occurring on histones, where they play an important role in transcriptional regulation, that is, a novel hallmark of epigenetic modification (Zhang et al., 2019). Nevertheless, followed studies have also identified K (L-la) modification on non-histone proteins(Gao et al., 2020; Zhang N. et al., 2021; Hagihara et al., 2021; Meng et al., 2021; Yang K. et al., 2022; Xiong et al., 2022). By contrast, the K (D-la) modification were mainly observed on non-histone proteins, most of which were enzymes involved in glycolysis, exerting negative feedback modulations on glycolytic output (Gaffney et al., 2020). Thus, here, unless specified, all the Kla and lactylation will suggest the K (L-la) instead of the K (D-la).

The discovery of protein lactylation opens a new era for deeper dissection of lactate metabolism and also provides a totally novel avenue for figuring out pathophysiological mechanisms of lactate in neoplastic and inflammatory diseases.

### 3.2 Writers and erasers

Protein acylations are evolutionarily conserved and reversible PTMs (Neumann-Staubitz et al., 2021). Rapid and dynamic changes in protein acylations have been shown to occur in response to enzymatic or non-enzymatic conditions (Zhao et al., 2010; James et al., 2017; James et al., 2018). Gaffney et al. proposed that MGO derived LGSH can directly lactylate lysine residues under a non-enzymatic acyl substitution condition, which was confirmed by the robust lactoyllysine generation after incubation of histone H4 with LGSH *in vitro* (Gaffney et al., 2020). However, most of histone acylations involve the so-called effectors, including writer and eraser proteins, that can exert the role of depositing and removing specific chromatin modifications, respectively. Some acylations, such as acetylation, β-hydroxybutyrylation, succinylation, and lactylation, shared the two effectors, the histone acetyltransferases and the histone deacetylases (HDACs), which possess their original function of acetylation or deacetylation (Hirschey and Zhao, 2015; Huang et al., 2018; Figlia et al., 2020).

p300, a classic histone acetyltransferase, was first reported to exert lactylation-writing function by Zhang and others in 2019 (Zhang et al., 2019). To confirm the activity of p300 as a histone Kla writer in cells, they overexpressed or knocked down p300 in HEK293T cells and observed elevation or reduction in histone Kla levels. Similarly, when p300 was knocked down in bone marrow-derived macrophages (BMDMs) from mice, lactate-induced histone Kla was considerably diminished (Cui et al., 2021). Subsequently, another study observed that silencing of p300 or its homologue CREB-binding protein (Cbp) by their specific siRNAs attenuated lactylated modification of high mobility group box-1 (Hmgb1) (Yang K. et al., 2022). This result can be recapitulated by the p300/Cbp inhibitor C646 (Yang K. et al., 2022). Using C646, another study also found a decreased trend of histone Kla levels in tumor-infiltrating myeloid cells (Xiong et al., 2022). Furthermore, Zhang et al. performed an *in-vitro* experiment by a cell-free, recombinant chromatin-templated histone modification and transcription assay, furtherly demonstrating the biogenesis of p53-dependent, p300-driven histone Kla(Zhang et al., 2019). Together, although indirect functions of p300/CBP cannot be excluded, these findings suggest that p300 and its homologue CBP are potential histone Kla writer proteins which act coordinately to regulate the biogenesis of histone Kla (Figure 1).

Borrowing from the discovery of lactylases, several studies evaluated zinc- and nicotinamide adenine dinucleotide (NAD)-dependent HDACs for their abilities of cleaving ε-N-l-lactyllysine marks (Jennings et al., 2021; Moreno-Yruela et al., 2022; Zessin et al., 2022; Zu et al., 2022). Jennings et al. presented the first report of the lactoyllys eraser, sirtuin 2 (SIRT2), which displayed strong capability of removing the L-lactoyllys from synthetic peptides related to pyruvate kinase M2 (PKM2) (Jennings et al., 2021). One additional study from Zu et al. confirmed SIRT2 functions as an efficient histone delactylase in multiple histone lactylation sites of synthetic histone peptides, purified histones and nucleosomes, and histones in neuroblastoma cells (Zu et al., 2022). Furtherly, HDAC1-3 and sirtuins (SIRT1-3) were identified as robust delactylases *in vitro* by Zhang and others. Of note, they highlight that not only K (L-la) but also K (D-la) can be removed by HDAC1-3. Subsequent analysis from overexpression and knockdown experiment using living cells in culture indicated that HDAC1 and HDAC3 but not HDAC2 play a specific role in delactylation of histone Kla(Moreno-Yruela et al., 2022). According to a separate study of Zessin et al., HDAC2, HDAC3, HDAC8, SIRT2 and SIRT3 were also potential delactylases *in vitro*. Similarly, they also confirmed the robust delactoylase, HDAC3, which harbored several-thousand-fold higher activity, compared to the major *in-vivo* delactoylase SIRT2(Zessin et al., 2022). Collectively, many histone deacetylases have the functions of delactylase (Figure 1), but their functional preference and switching mechanism remains a mystery.

### 3.3 The modification sites

Protein acylations have been recognized for over 50 years. It is originally observed on histone proteins, exerting a critical role in modulating transcriptions (Phillips, 1963; Allfrey et al., 1964). Follow-up studies showed that many other proteins can also be acylated on lysine residues. Here, we summarize the lactylation landscape, including histone and non-histone Kla sites currently identified in multiple organisms, as below.

#### 3.3.1 Histone lactylation sites

In 2019, Zhang and others identified 26 and 18 histone Kla sites on H3, H4, H2A and H2B of core histones from human HeLa cells and mouse BMDMs, respectively (Zhang et al., 2019). Subsequently, several groups reported many other histone Kla sites from mouse brain tissues (Hagihara et al., 2021), protozoan parasite Trypanosoma brucei (*T. brucei.*) (Zhang N. et al., 2021), Botrytis cinerea (*B. cinerea*) (Gao et al., 2020) and *Oryza sativa* (Meng et al., 2021). Hagihara et al. identified 16 histone Kla sites from the prefrontal cortex of social defeated mouse (Hagihara et al., 2021). They highlighted the increase of histone H1 lactylation in response to the social defeat stress. According to the study of Zhang et al., there are also 16 histone Kla sites distributed across all canonical and variant histones in *T. brucei*. (Zhang N. et al., 2021). Intriguingly, they found that all 16 Kla sites were associated with other PTMs. Other interested research communities include those studying *B. cinerea* and *Oryza sativa*, which identified 6 and 14 histone Kla sites, respectively (Gao et al., 2020; Meng et al., 2021). Of note, these results were obtained from different species and were induced in a spectrum of physiological and pathological conditions. Therefore, we speculate that the discrepancy in histone Kla sites might be due to the differences in species or spatial-temporal dynamics (Table 1).

**TABLE 1:** A complete list of the identified histone lactyllysine sites in cross-species samples.

Histone	Biological sample	Species	Lactyllysine sites	References
**Pan**	A549	Human	-	Jiang et al. (2021a)
	H1299	Human	-	Jiang et al. (2021a)
	OCM1	Human	-	Yu et al. (2021)
	CRMM1	Human	-	Yu et al. (2021)
	MCF-7	Human	-	Zhang et al. (2019)
	Brain tissue (AD)	Human, Mouse	-	Pan et al. (2022)
	OG2 mouse embryonic fibroblast	Mouse	-	Li et al. (2020)
	BMDM	Mouse	-	Zhang et al. (2019), Irizarry-Caro et al. (2020), Cui et al. (2021), Xiong et al. (2022)
	TAM (B16-bearing mice)	Mouse	-	Zhang et al. (2019)
	TAM (LLC-bearing mice)	Mouse	-	Zhang et al. (2019)
	Blastocyst	Mouse	-	Yang et al. (2021)
	Macroglia (AD mice)	Mouse	-	Pan et al. (2022)
**H3**	Hela	Human	9, 18, 23, 27, 79	Zhang et al. (2019)
	PIG1	Human	18^▲^	Yu et al. (2021)
	OCM1	Human	18^▲^	Yu et al. (2021)
	MEL290	Human	18^▲^	Yu et al. (2021)
	92.1	Human	18^▲^	Yu et al. (2021)
	OMM1	Human	18^▲^	Yu et al. (2021)
	MUM2B	Human	18^▲^	Yu et al. (2021)
	CRMM1	Human	18^▲^	Yu et al. (2021)
	CRMM2	Human	18^▲^	Yu et al. (2021)
	CM2005.1	Human	18^▲^	Yu et al. (2021)
	Peripheral blood mononuclear cell	Human	18^▲^	Chu et al. (2021)
	Ocular melanoma tissue	Human	18	Yu et al. (2021)
	Ishikawa cells	Human	18	Yang et al. (2022b)
	AB2.2	Mouse	18^▲^	Tian and Zhou, (2022)
	OG2 mouse embryonic fibroblast	Mouse	18^▲^	Li et al. (2020)
	BMDM	Mouse	14, 18^▲^, 23, 27, 56	Zhang et al. (2019), Sun et al. (2021b), Xiong et al. (2022)
	MDSC (co-cultured with MC38)	Mouse	18^▲^	Xiong et al. (2022)
	Macroglia (AD mice)	Mouse	18	Pan et al. (2022)
	Brain tissue (AD)	Mouse	18	Pan et al. (2022)
	Blastocyst tissue	Mouse	18^▲^, 23^▲^	Yang et al. (2021)
	Endometrial tissue	Ovine	18^▲^	Yang et al. (2022b)
	BSF of *T. brucei*	Protozoan Parasite	24, 33, 62	Zhang et al. (2021b)
	*B.cinerea*	Plant fungal pathogen	123	Gao et al. (2020)
	Shendao 529 rice	*Oryza sativa*	9, 14, 18, 56	Meng et al. (2021)
**H4**	Hela	Human	5, 8, 12, 16, 31, 77, 91	Zhang et al. (2019)
	Recombinant histone H4	Human	31	Gaffney et al. (2020)
	MCF-7	Human	5	Zhang et al. (2019)
	Brain tissue (AD)	Human, Mouse	12	Pan et al. (2022)
	BMDM	Mouse	8, 12, 31, 91	Zhang et al. (2019)
	AB2.2	Mouse	8, 12	Tian and Zhou, (2022)
	Macroglia (AD mice)	Mouse	12^▲^	Pan et al. (2022)
	BSF of *T. brucei*	Protozoan Parasite	78	Zhang et al. (2021b)
	Shendao 529 rice	Oryza sativa	5, 8, 16, 31	Meng et al. (2021)
**H2A**	Hela	Human	11, 13, 115	Zhang et al. (2019)
	BMDM	Mouse	11, 115	Zhang et al. (2019)
	BSF of *T. brucei*	Protozoan Parasite	5, 21, 116	Zhang et al. (2021b)
	*B.cinerea*	Plant fungal pathogen	5, 23	Gao et al. (2020)
**H2A.Z**	BSF of *T. brucei*	Protozoan Parasite	32, 36, 44, 165	Zhang et al. (2021b)
**H2B**	Hela	Human	5, 11, 15, 16, 20, 23, 43, 85, 108, 116, 120	Zhang et al. (2019)
	BMDM	Mouse	5, 11, 15, 16, 20, 85, 108	Zhang et al. (2019)
	BSF of *T. brucei*	Protozoan Parasite	5, 97	Zhang et al. (2021b)
	*B.cinerea*	Plant fungal pathogen	15, 48, 122	Gao et al. (2020)
	Shendao 529 rice	Oryza sativa	41, 60, 66, 114,136, 144	Meng et al. (2021)
**H2Bv**	BSF of *T. brucei*	Protozoan Parasite	8, 20, 28	Zhang et al. (2021b)
**H1**	Brain tissue (social defeat stress)	Mouse	Unknown	Hagihara et al. (2021)

MDSC, myeloid-derived suppressor cells; BSF, the bloodstream form; ^▲^Functional sites have been validated.

#### 3.3.2 Non-histone lactylation sites

With the development of high-throughput technologies (i.e., HPLC-MS/MS), other proteins, except core histones above, distributed in the nucleus, cytoplasm, mitochondria, ribosome and cell membrane were also found harboring massive and diverse Kla sites. In 2020, Gaffney et al. identified 350 lactoyllysine modified proteins in HEK293T cells, most of which were enzymes involved in glycolysis (Gaffney et al., 2020). Another study reported 63 lactylated proteins in mouse brain tissues (Hagihara et al., 2021). Among them, twelve proteins were supposed to be regulated by social defeat stress. A total of 273 Kla sites from 166 lactylated proteins were identified from *B. cinerea*. Eighty two Kla sites were from 43 ribosomal proteins, suggesting the critical roles of lactylation in cytoplasmic translation (Gao et al., 2020). Subsequently, in the analysis of *T. brucei.*, Zhang et al. recognized 387 Kla sites from 257 proteins. Further analysis revealed that heat shock protein 90 (HSP90), with 14 independent lactylated lysine residues, was the most extensively lactylated proteins in *T. brucei.* (Zhang N. et al., 2021). Similar findings were also reported by Meng and others, exhibiting 638 Kla sites from 342 proteins in Rice (*Oryza sativa*) Grains (Meng et al., 2021) (Table 2). On the basis of the hundreds of lactylated proteins/sites, Gao et al. summarized that 50% of the lactylated proteins have a molecular weight higher than 40k Dalton and 40% of the lactylated sites are located in regions with ordered secondary structures, presenting a preference in alpha-helix structure than beta-strand or disordered (Gao et al., 2020).

**TABLE 2:** Distribution and functional profile of non-histone lactylated proteins in cross-species samples.

Biological sample	Species	Pro no.	Site no.	Subcellular location	Functional profile	References
HEK293	Human	350	-	Nucleus 23.2%	**Most massively modified on:** GLO1, GLOD4 **Mainly enriched in:** carbon metabolism and glycolysis	Gaffney et al. (2020)
				Cytosol 49.9%		
				Mitochondria 5.5%		
Brain tissue	Mouse	47	-	Nucleus 20.0%	**Mainly enriched in:** extracellular exosome, nucleosome	Hagihara et al. (2021)
				Cytosol 22.2%		
				Mitochondria 6.7%		
RAW264.7	Mouse	1	-	Nucleus	**Modified on:** Hmgb1 **Function:** promoted Hmgb1 translocation from the nucleus to the cytoplasm and its release via exosome secretion	Yang et al. (2022a)
BMDM, MDSC (co-cultured with MC38)	Mouse	1	2	Nucleus	**Modified on:** Mettl3 (K281, K345)	Xiong et al. (2022)
					**Function:** enhanced the capture of m^6^A-modified RNA	
BSF of *T. brucei*	Protozoan Parasite	251	371	Nucleus 38.0%	**Most massively modified on:** HSP90	Zhang et al. (2021b)
				Cytosol 35.0%	**Mainly enriched in:** the cytosolic large ribosomal subunit, carbohydrate catabolism, translation elongation factor activity	
				Mitochondria 11.0%			
*B.cinerea*	Plant fungal pathogen	163	267	Nucleus 36.0%	**Mainly enriched in:** proteins associated with ribosome, fungal pathogenicity regulated proteins	Gao et al. (2020)
				Cytosol 25.0%		
				Mitochondria 27.0%		
Shendao 529 rice	*Oryza sativa*	339	630	Nucleus 9.9%	**Most massively modified on:** Glyoxalase family protein, α-amylase/trypsin inhibitor 2, nonspecific lipid transfer protein 1 **Mainly enriched in:** starch Biosynthesis, central carbon metabolism	Meng et al. (2021)
				Cytosol 33.0%		
				Mitochondria 9.1%		
				Chloroplast 38.3%		

GLOD4, glyoxalase domain-containing protein four; Pro No., the number of non-histone lactylated protein; Site No., the number of non-histone lactyllysine site.

In addition to the aforementioned high-throughput MS investigations, some lactylated proteins were also found playing critical roles in the cellular activity regulations. Yang et al. found that the nuclear protein Hmgb1 can be directly lactylated in a p300/CBP-dependent mechanism upon lactate stimulation, which led to its translocation from the nucleus to cytoplasm (Yang K. et al., 2022). This report, however, did not reveal the specific lactylation sites in Hmgb1. More recently, another study of Xiong and others identified two Kla sites of methyltransferase 3 (Mettl3), including K281 and K345, which locate in the first CCCH-type zinc fingers domain and the linker, respectively (Xiong et al., 2022). The lactylation of these two sites on Mettl3 enhanced its capability in capturing N6-methyladenosine (m^6^A)-modified RNA. All studies mentioned above suggest that not only core histones but also proteins with a diversified cellular distribution can be lactylated directly, making the regulation in cellular activities widely available (Table 2).

Identification of the exact sites of Kla is an essential step for understanding the molecular mechanisms of lactylation. Jiang et al. first developed an on-line application of the Kla prediction model, named FSL-Kla(Jiang P. et al., 2021). It enabled further optimization for experimental validation compared to labor-intensive and time-consuming ways. The FSL-Kla, freely accessible for academic research at *http://kla.zbiolab.cn/* , not only is a cutting-edge tool for Kla site profile but also could generate candidates for further experiments.

## 4 Physiological and pathological roles of lactylation

### 4.1 Lactylation and cell fate determination

**FIGURE 2:**
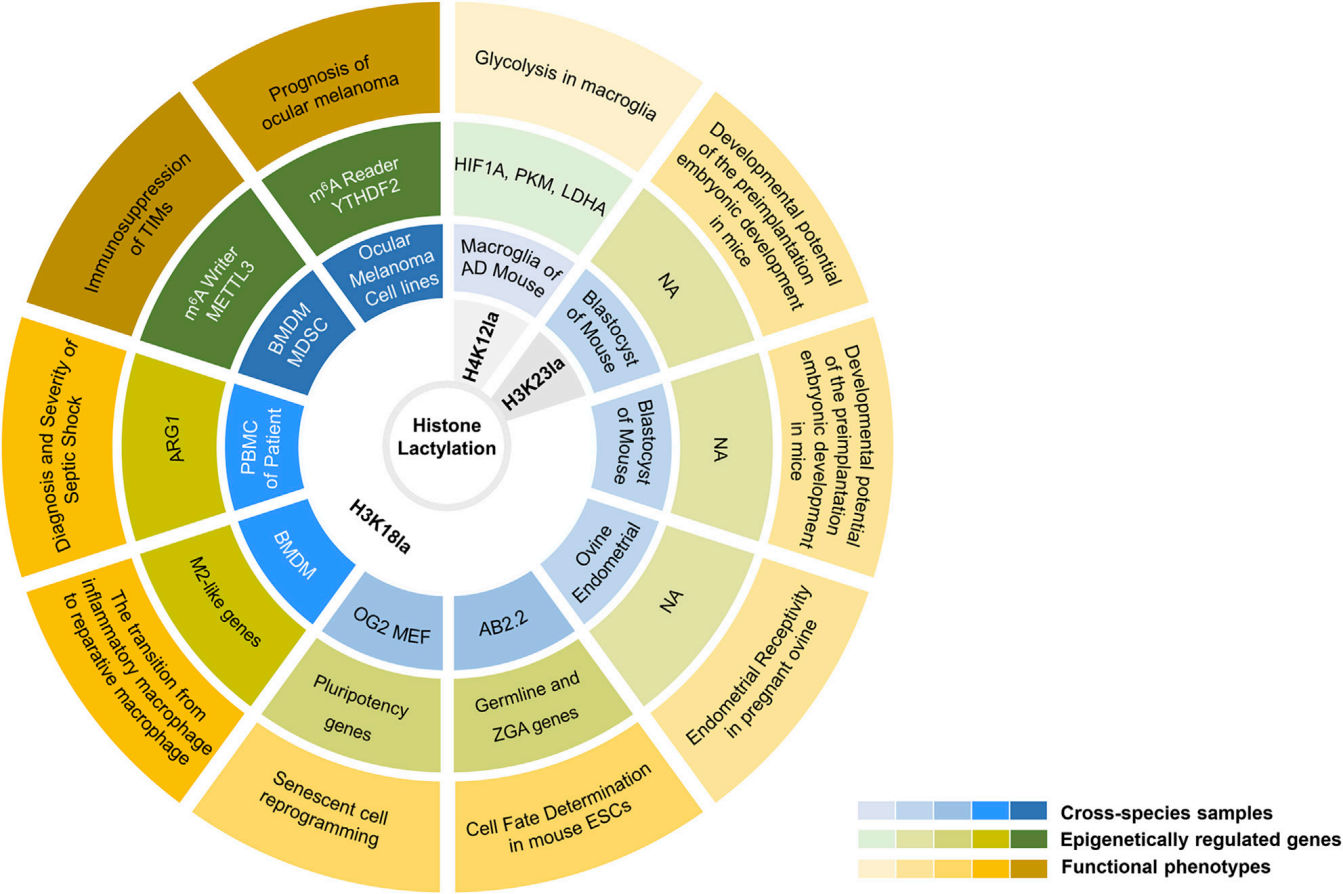
An elaborate integrated figure presenting biological functions of histone lysine lactylation in available studies. From outside to inside: functional phenotypes, epigenetically regulated genes, cross-species samples, and histone lactylation sites. From shallow to deep: histone lactylation in neuropsychiatric disorders, embryonic development, cell fate determination, inflammation and neoplastic disease. NA, not available.

Cell fate determination is a multistep process that is finely regulated during the complicated life activities (Haran and Lenka, 2019; Zechner et al., 2020; Balsalobre and Drouin, 2022). This process not only relies on the precise regulation of transcription levels but also on multi-level of epigenetic and metabolic regulations (Baksh and Finley, 2021). Recently, Li and others revealed an epigenome-metabolome-epigenome signalling cascade during the induction of pluripotency in stem cells (Li et al., 2020). During somatic reprogramming, the maternal Gli-like transcription factor 1 (Glis1) enables the closure of somatic genes at early stages while opens chromatin at glycolytic genes, including hexokinase 2 (*Hk2*), phosphoglycerate kinase 1 (*Pgk1*), phosphofructokinase, liver type (*Pfkl*), *Pkm1/2*, enolase 1 (*Eno1*), *Ldha*, etc. Followed by the metabolic remodeling, Glis1 quickly activates glycolysis, thus enhancing histone lactylation (H3K18la), opening pluripotency genes, promoting senescent cell reprogramming and improving the genome stability of the induced pluripotent stem cells. Similarly, another study observed the upregulation of germline and cleavage embryo genes in mouse embryonic stem cells after the accumulation of histone Kla(Tian and Zhou, 2022). Further analysis revealed that the remarkably increased deposition of H3K18la at the transcription start site might be responsible for the sequential activation of germline and zygotic genome activation genes. According to the above two, it is convincible that histone Kla, especially for H3K18la, plays a pivotal role in shaping the process of cell fate decisions. Of note, during somatic reprogramming, H3K18la tended to be highly enriched at the promoters of pluripotency genes, such as Octamer-binding transcription factor 4 (*Oct4*), spalt like transcription factor 4 (*Sall4*) and MYCN proto-oncogene (*Mycn*), which subsequently upregulated their expressions (Li et al., 2020). In contrast, the expression of pluripotent genes, including *Oct4*, Nanog homeobox (*Nanog*), and SRY-box transcription factor 2 (*Sox2*), were not affected upon lactate supplementation in mouse embryonic stem cells (Tian and Zhou, 2022). That is, lactate strengthened H3K18la without affecting pluripotency of mouse embryonic stem cells. The underlying mechanism of the context-dependent epigenetic regulation of transcription by histone lactylation is worth further in-depth investigation (Figure 2).

### 4.2 Lactylation and embryonic development

During embryogenesis, dynamic changes of PTMs, such as methylation, acetylation, phosphorylation, ubiquitylation, sumoylation, etc., play a critical role in zygotic genome activation and cell lineage specification (Torres-Padilla et al., 2007; Rossetto et al., 2012; Sabari et al., 2017; Uckelmann and Sixma, 2017; Liu et al., 2018; Jambhekar et al., 2019; Liu et al., 2020). Lactylation, identified by Zhang et al., in 2019, is also one of those (Zhang et al., 2019). In 2021, Yang et al. first provided a dynamic landscape of H3K23la, H3K18la and pan histone lactylation in oocytes and pre-implantation embryos of mouse (Yang et al., 2021). According to their study, the pan histone lactylation, H3K23la and H3K18la in oocytes, pre-implantation and the early cleavage stage embryos maintain a low level under normal conditions, which is in contrast to their sharp increases in blastocyst stage embryos. Further analysis revealed that the oxygen concentration in the culture environment was responsible for the changes in histone lactylation levels of different periods of embryos (Yang et al., 2021). Normally, hypoxia increases glycolysis, along with a growing accumulation of endogenous lactate, thus leading to increased histone Kla levels (Zhang et al., 2019). Interestingly, contrary to this, long-term hypoxia after fertilization dampens the expression of *Ldha*, thereby maintaining a low level of histone lactylation and eventually leading to a detrimental effect on developmental potential of the preimplantation embryonic development. In addition to influencing embryo development, histone lactylation was found playing a pivotal role in uterine remodeling (Yang Q. et al., 2022). Yang et al. observed a robust increase of H3K18la in the endometrium of pregnant ovine, compared to a sharp decrease of H3K18la in the endometrium undergoing pregnancy failure, which highlight the critical role of H3K18la in maintaining endometrial receptivity. Mechanistically, lactate, derived from enhanced glycolysis, promotes histone lactylation in endometrium via the ovine maternal-fetal interface and enables epigenetic regulation of redox homeostasis and apoptotic balance, eventually shaping the process of uterine remodeling and embryo implantation (Figure 2).

### 4.3 Lactylation in inflammation and immune response

In spite of being recognized as a simple metabolic waste product for a long time, lactate is now revisited as a signaling molecule in immunomodulatory effects. Increasing evidence indicates the pivotal role of lactate in acute, chronic inflammation processes, where it acts as a pro- or anti-inflammatory mediator, exerting differential effects depending on the metabolic status and cell types (Certo et al., 2021; Manosalva et al., 2021). In 2019, Zhang et al. revealed for the first time that the lactylation of histone lysine residues, a novel epigenetic modification, served as a mechanistical key linker between lactate and immunomodulatory process, which opened a new era for deeper investigating the mechanisms of lactate in inflammation and immune response (Zhang et al., 2019).

#### 4.3.1 Acute infections and sepsis

To our knowledge, macrophage activation is a key event in the process of acute inflammation, especially in septic shock (Benoit et al., 2008; Karakike and Giamarellos-Bourboulis, 2019). M1, a pro-inflammatory macrophage phenotype is always associated with active aerobic glycolysis, resulting in a massive lactate accumulation (Galván-Peña and O'Neill, 2014). Whereas high levels of arginase 1 (ARG1) in anti-inflammatory M2 macrophages enable the production of ornithine to facilitate wound healing and cellular homeostasis (Van den Bossche et al., 2012). Zhang et al. revealed that treatment of BMDMs with lipopolysaccharide (LPS) and interferon-γ, that is M1 activation, robustly increased intracellular lactate and histone Kla levels. Whereas both the levels of lactate and global histone Kla were decreased after treatment of the Ldha-specific inhibitor GNE-140 during M1 polarization, suggesting that the endogenous production of lactate via glycolysis is the source for histone lactylation (Zhang et al., 2019). Alternatively, using such a bacterial infection model, Zhang and others also observed that the expression of inflammatory response and homeostasis genes was undergoing dynamic changes. The inflammatory response genes (e.g. nitric oxide synthase 2 (*Nos2*), interleukin 6 (*Il6*), tumor necrosis factor (*Tnf*), etc.) were upregulated in the early 4 h and steadily declined at later time points during M1 polarization (Zhang et al., 2019). Meanwhile, the homeostatic M2-like genes (e.g. Arg*1*, matrix metallopeptidase 9 (*Mmp9*), reticulon four receptor (*Rtn4r*), etc.) were increased at later time points after M1 polarization, indicating a late-phase switch to a more homeostatic phenotype. Of note, the induction of these M2-like genes displayed a temporal association with accumulated lactate and elevated histone Kla (especially for H3K18la). On the basis of these data, they proposed a ‘lactate timer’ in macrophages, which turns on homeostatic M2-like gene expression in the context of high levels of histone Kla and eventually repairs collateral damage incurred by the host during infection (Zhang et al., 2019) (Figure 2).

Similarly, in the analysis of peripheral blood mononuclear cells from septic shock patients, Chu et al. observed a positive correlation between H3K18la and ARG1 mRNA level (Chu et al., 2021). That is, H3K18la can mediate inflammatory cytokine and ARG1 overexpression, which subsequently stimulates the anti-inflammatory function of macrophages in sepsis (Figure 2). It is worth noting that, however, Dichtl et al. argued these interpretations (Dichtl et al., 2021). Their results questioned the ‘lactate timer’, arguing that except for Arg*1* gene, they failed to reproduce a clear association between inflammatory stimulation (LPS-induced), lactate, lactylation, time and M2-like gene expression as described by Zhang and others (Zhang et al., 2019). According to them, LPS-induced Arg*1* was instead dependent on autocrine-paracrine Il6 production, Il6 receptor, and signal transducer and activator of transcription 3 (Stat3) signal transduction (Dichtl et al., 2021). Indeed, there are still some discrepancies in the relationship between histone Kla and *ARG1* expression. Additional studies are needed to clarify these controversial findings.

In addition to serving as an anti-inflammatory mediator, lactate-derived lactylation may also plays a positive role in promoting the infection process under specific circumstances. A recent study reported that lactate administration strongly increased serum Hmgb1 levels and decreased survival rate of septic mice (Yang K. et al., 2022). Mechanistic analysis revealed that lactate can directly induce nuclear Hmgb1 lactylation in mouse macrophages in a p300/Cbp-dependent manner. The lactylated Hmgb1 was translocated from the nucleus to the cytoplasm and subsequently released via exosome secretion. These events together impaired the endothelium integrity and vascular permeability, leading to an adverse outcome of polymicrobial sepsis.

Another clinical study by Chu et al. suggested that H3K18la can serve as a potential biomarker for the diagnosis and predict the severity of septic shock (Chu et al., 2021). According to them, pan-protein lactylation in peripheral blood mononuclear cells were significantly increased in patients with shock and other critical illness. Particularly, the mean level of H3K18la was the highest in septic shock patients, compared to non-septic shock patients and healthy controls (Chu et al., 2021). In septic shock patients, high levels of H3K18la also exhibited a closely positive correlation with inflammatory cytokines and parameters, including IL6, IL10 and procalcitonin (Chu et al., 2021) (Figure 2). Together, these studies presented above indicate that lactate-derived lactylation, either histone or non-histone proteins, may strongly contribute to the process of acute inflammation.

#### 4.3.2 Chronic inflammation and fibrosis

Macrophage activation in the non-specific, chronic inflammation was also found being associated with protein lactylation. Irizarry-Caro et al. found that in dextran sulfate sodium-induced inflammatory colitis model, mice that lack B-cell adapter for PI3K (*Bcap*) in macrophages suffered from more severe intestinal inflammation and tissue injury than that in wild type control mice (Irizarry-Caro et al., 2020). Mechanistic analysis revealed that the deficiency of *Bcap* dampened the reparative phase of macrophage response, with low levels of glycolysis, lactate, protein lactylation and transcription of reparative genes, including Arg*1*, Kruppel like factor 4 (*Klf4*), and *Mmp9*. Exogenous lactate administration, conferring high levels of histone Kla, enabled the transition of macrophages from an inflammatory state to a reparative state, thereby rescuing Bcap-deficient phenotype (Irizarry-Caro et al., 2020). In a separate study, lactic acid derived from *Saccharomyces cerevisiae* (*S. cerevisiae*) also showed anti-inflammatory activity in dextran sulfate sodium-induced colitis mice (Sun S. et al., 2021). Similarly, after lactic acid treatment, the decreased pro-inflammatory cytokines (e.g., Tnfα, Il6, and Il1β), the suppressed M1 macrophage polarization and the upregulated H3K18la levels were observed. Altogether, the study of Sun et al. also indicate that lactic acid derived from *S. cerevisiae* mediated histone H3K18 lactylation, promoting the shift from M1-to M2-like profile of macrophages, which eventually improved histological damage, increased mucosal barrier, and decreased intestinal immune response (Sun S. et al., 2021).

There are, of course, cases of pro-inflammation during macrophage activation in the non-specific, chronic inflammation. Cui et al. presented a pivotal role of protein lactylation in the profibrotic activity of alveolar macrophages (Cui et al., 2021). In bleomycin-induced or transforming growth factor-β1-induced lung fibrosis models, considerable lactate was observed in lung myofibroblasts. They showed that lactate derived from the increased glycolysis in the alveolar macrophages from fibrotic lungs markedly upregulated the expression of profibrotic genes, such as Arg*1* and vascular endothelial growth factor A (*Vegfa*) by inducing histone lactylation at promoters of these genes (Cui et al., 2021).

Of particular note, central nervous system-intrinsic microglia are highly similar to macrophages, which can also be regulated by protein lactylation. An updated study showed increased levels of Pan-lysine lactylation (Pan Kla) and H4K12la in both Alzheimer’s disease (AD) mouse models and human AD brain tissues (Pan et al., 2022). The elevated H4K12la modification in amyloid-β (Aβ) plaque adjacent microglia activated the transcription of multiple genes encoding glycolytic enzymes, by which it ‘locked in’ the metabolic rewiring of microglia in AD and eventually drove the pro-inflammatory microglial activation by a positive feedback loop, termed as glycolysis/H4K12la/Pkm2 (Pan et al., 2022) (Figure 2).

In summary, these observations can indeed illustrate the critical role of protein lactylation during macrophage/microglia activation in the non-specific, chronic inflammation. Further studies may mainly aim at distinguishing their regulatory, compensatory or feedback mechanisms under physiological and pathological conditions.

### 4.4 Lactylation in neoplastic disease

The rewiring of energy metabolism is necessary for sustaining the tumor growth and proliferation (Ippolito et al., 2019; Sun L. et al., 2021). The Warburg effect and the massive accumulation of lactate also imply that cancer is a set of metabolic disease (Martínez-Reyes and Chandel, 2021). Currently, roles of lactylation in the regulation of several processes in neoplastic disease have been documented. Yu et al. found an increasing level of histone lactylation (H3K18la) in both human ocular melanoma tissues and cell lines, which was described as a factor associated with poor prognosis in ocular melanoma patients (Yu et al., 2021). Further analysis revealed that the enrichment of H3K18la at the promoter of an m^6^A reader YTH N6-methyladenosine RNA binding protein 2 (*YTHDF2*) promoted the expression of *YTHDF2* and further impaired mRNA stability of period circadian regulator 1 (*PER1*) and tumor protein p53 (*TP53*), leading to an aggressive proliferation and migration of ocular melanoma cells. This study revealed a novel mechanism of histone Kla in tumorigenesis and described a crosstalk between histone lactylation and RNA m^6^A methylation (Figure 2). In another study, lactate played a role in inhibiting glycolysis and maintaining mitochondrial homeostasis in non-small cell lung cancer (NSCLC) cells, presenting down-regulated transcription of *HK1* and *PKM* as well as up-regulated of succinate dehydrogenase complex flavoprotein subunit A (*SDHA*) and isocitrate dehydrogenase three non-catalytic subunit gamma (*IDH3G*) (Jiang J. et al., 2021). When treated with lactate, histone lactylation increased and enriched at the promoter of genes encoding these metabolic enzymes in NSCLC cells, directly acting on cellular metabolism and eventually modulating proliferation and migration of NSCLC (Jiang J. et al., 2021).

Additionally, protein lactylation also plays a critical role in tumor-infiltrating immune cells (Zhang et al., 2019; Xiong et al., 2022). According to the study of Zhang et al., higher levels of histone Kla were observed in tumor-associated macrophages (TAM) isolated from B16F10 (melanoma) or LLC1 (lung cancer) tumor-bearing mice (Zhang et al., 2019). With high levels of histone Kla, tumor-associated macrophages presented a tumor-promoting M2 phenotype, thereby strongly contributing to the formation and progression of tumors. Another up-dated study revealed that lactate derived from the tumor microenvironment drove the transcription of immunosuppressive genes in tumor-infiltrating myeloid cells via modulating Mettl3 (Xiong et al., 2022). On one hand, lactate increased the transcription of *Mettl3* by enhancing the enrichment of H3K18la at the promoter region of *Mettl3*; On the other hand, direct lactylation of Mettl3 at K281 and K345 enhanced its capability of capturing m^6^A-modified RNA. The combination of the two collectively contributed to the enhanced translation efficiency of Janus kinase 1 (Jak1) mRNA and the subsequent activation of Jak-Stat3 pathway (Xiong et al., 2022). Similar to the studies of Yu et al., these results reproduced the crosstalk between protein lactylation and RNA m^6^A modification and robustly confirmed the negative functions of protein lactylation in tumor-infiltrating immune cells (Figure 2).

### 4.5 Lactylation in neuropsychiatric disorders

Neuronal activities throughout the brain have a great deal of metabolic demands (Yellen, 2018). Lactate and glycolysis have been implicated in multiple regulations of physiological or pathological neuronal activities, including neuronal plasticity (Yang et al., 2014), neuron-glia interactions (Tang et al., 2014), neuroimmune communication and many other neuropsychiatric disorders (Xie et al., 2016; Li et al., 2018; Kaushik et al., 2019). Recently, Hagihara et al., for the first time, identified protein lactylation which can be stimulated by the neural-activity-induced lactate in mouse brain (Hagihara et al., 2021). According to them, histone lactylation in brain cells can be up-regulated by neural excitation and social defeat stress. Further analysis found a robust increase of protein lactylation in the prefrontal cortex from mice received anxiogenic stimulation, along with the upregulation of Fos proto-oncogene (*Fos*) (Hagihara et al., 2021). High Kla levels subsequently resulted in the decreased social interaction and the increased anxiety-like behaviors of mice. These results confirm the existence of protein lactylation in brain, being regulated by neuronal activity and social defeat stress. Exploring the landscape of protein lactylation and the regulatory roles of protein lactylation in etiology and pathophysiology of neuropsychiatric disorders attracts great interest in the field.

More recently, Pan et al. first identified high levels of pan-Kla and histone Kla in both the prefrontal cortex and hippocampus of AD mouse models and postmortem brain tissues of AD patients (Pan et al., 2022). Their observations highlight the significant increase of H4K12la in Aβ plaque-adjacent microglia. Analysis of the underlying mechanisms revealed that H4K12la enriched at promoters of several glycolytic genes, including *Pkm2*, and subsequently promoted the formation of a viciously metabolic loop, comprising activated glycolysis, H4K12la, and Pkm2, which collectively drove pro-inflammatory microglial activation and neuroinflammation in AD. Taking this a step further, they interrupted this loop by inhibiting Pkm2 pharmaceutically or knocking out *Pkm2* genetically, with an observation of compensatory expression of *Pkm1* and a metabolic transition from glycolysis toward oxidative phosphorylation (OXPHOS), which eventually reduced Aβ burden and improved spatial learning and memory in AD mouse models (Pan et al., 2022). Overall, this study, for the first, proposed a microglial glycolysis/H4K12la/Pkm2 positive feedback loop that was responsible for the progression of AD, concurrently indicating a potential therapeutic target for AD (Figure 2).

## 5 Currently open issues

### 5.1 Controversy in “lactylgenesis” machinery

There are currently two mechanistic hypotheses for the explanation of ‘lactylgenesis’, which were implicated by l-lactate and d-lactate, respectively (Zhang et al., 2019; Gaffney et al., 2020). l-lactate [with (S) configuration] is the predominant enantiomer, with a physiological serum concentration of 1–2 mM and reaching up to 40 mM in some tumor cells (Walenta et al., 2000). d-lactate [with (R) configuration], a structural isomer of l-lactate, is primarily produced from MGO by the glyoxalase pathway (Landro et al., 1992; Sousa Silva et al., 2013; de Bari et al., 2019), presenting a lower serum concentration (11–70 nM), compared to l-lactate (Ewaschuk et al., 2005; Levitt and Levitt, 2020). In 2019, Zhang et al. first proposed that l-lactate, acting as a precursor, can be incorporated into histone K (L-la) (Zhang et al., 2019). Taking the chemically unfavorable binding between lactate and free amines of histones into consideration, they speculated and confirmed that L-lactyl-CoA, an activated form of l-lactate, might act as a direct substrate for enzymatically transferred lactyl groups to lysine residues on histones (Figure 1). Despite offering innovatively bright spots, several limitations in this study invite following skepticism. One obvious question is the lack of widely detection of L-lactyl-CoA in biological samples. Only one study, up to now, confirmed the existence of L-lactyl-CoA in both human HepG2 cells and heart and muscle tissues of mouse, but in a low picomole abundance (Varner et al., 2020). Another open question should be focused on the undefined putative writer and eraser of protein lactylation, which play the critical role in their mechanistic hypothesis. According to the updated results, p300 and HDAC1/3 were confirmed to be highly efficient enzymes mediating the process of histone lactylation and delactylation (Zhang et al., 2019; Cui et al., 2021; Jennings et al., 2021; Moreno-Yruela et al., 2022; Xiong et al., 2022; Yang K. et al., 2022; Zessin et al., 2022; Zu et al., 2022). However, there is no evidence suggesting that which step p300 and HDAC1/3 directly involved in the process from l-lactate, L-lactyl-CoA to lactyllysine, meaning that the putative enzymes in this enzymatic mechanism remain unclear.

One additional hypothesis described by Gaffney et al., albeit less referred by the following studies, was a clear mechanism for K (D-la) using lactylglutathione as the direct substrate (Gaffney et al., 2020) (Figure 1). This hypothesis presents a non-enzymatic process from MGO, LGSH to lactoyllysine, whereas they believed that K (D-la) should be tightly regulated at the “eraser” side. According to them, lactoyllysine modifications would presumably accumulate in the absence of a dedicated “eraser” protein (Jennings et al., 2021). In contrast to the hypothesis of Zhang et al., almost all details in mechanism of [K (D-la)] have been rigorously validated, including both the production and non-enzymatic transfer of LGSH. We speculate that a key reason for less attention of their theory is that they link lactylation to d-lactate, whose physiological concentration (in the nanomolar range) is typically thousands of times lower than l-lactate (in the millimolar range) in cells.

Collectively, additional studies are needed to address these complicated issues and establish the relationships between different mechanisms of lactylation. Future work aiming at seeking the enzymes involved in the production and transfer of L-lactyl-CoA will provide new insights into mechanisms of protein lactylation.

### 5.2 Lactylation, transcriptional regulation and macrophage polarization

Findings of lactylation offer important implications, which highlight the profound effects of lactate, a single metabolite, on cellular functions. Currently functional work of lactylation mainly focused on its functions in transcriptional regulation and macrophage polarization. To our knowledge, macrophages, presenting pro-inflammatory (M1-like) or anti-inflammatory (M2-like) characteristics, respond to metabolic alterations that correspond to their specific functions (Galván-Peña and O'Neill, 2014). In 2014, Colegio et al. first reported that tumor-cell-derived lactic acid induced the expression of *Vegf*, Arg*1* and other M2-associated genes, leading to a switch of M1-to M2-like macrophages in tumor (Colegio et al., 2014). However, the mechanisms whereby lactate promotes macrophage polarization remain unclear. In 2019, Zhang and others first described the kinetics of lactylation in the context of macrophage inflammation (Zhang et al., 2019). In their study, LPS and interferon-γ triggered the activation of macrophages and exacerbated inflammation, along with elevated levels of intracellular lactate, histone lactylation and expression of inflammatory genes.

Intriguingly, Zhang et al. have noticed that the increased expression of inflammatory genes induced by LPS/interferon-γ preceded histone Kla and occurred independently (Zhang et al., 2019). Indeed, they found a slower increase of histone Kla, which was confirmed tightly correlating with the upregulation of homeostatic (termed M2-like) genes, including the classic Arg*1*. After that, follow-up studies rapidly confirmed their findings in alveolar macrophage (Cui et al., 2021), BMDMs (Irizarry-Caro et al., 2020; Sun S. et al., 2021) and human peripheral blood mononuclear cells (Chu et al., 2021), respectively. In these studies, despite absence of kinetic models for lactylation, the elevated histone Kla is in all cases accompanied by the upregulation of homeostatic genes, especially Arg*1*, indicating a shift from the M1-to the M2-like profile of macrophage. In a separate study, however, the author argued that histone Kla was a consequence rather than a cause of macrophage polarization (Dichtl et al., 2021). They highlight that histone Kla occurs concurrently with an Il6-and Arg1-dependent metabolic switch under inflammatory stress. In some cases, of course, excessive accumulation of lactate led to the increased deposition of Kla marks on tissue repair genes, thus promoting their overexpression and presenting collateral damage following an unrestrained inflammatory response, which is responsible for the role of histone Kla in pro-inflammatory processes (Cui et al., 2021).

Whether histone Kla governs the transition from inflammatory macrophages to reparative macrophages remains elusive. First, we have no idea as yet how the promoters of M2-like genes are preferentially modified by histone Kla during macrophage activation. Certainly, this may be ascribed to the identification of writer and eraser mentioned by enzymatic mechanism of lactylation. Second, it is still unknown what is the bottom line of lactate concentration that can drive epigenetic regulations via histone Kla. Furthermore, whether lactate concentration could be used as cut-off values accounting for the functional transition of histone Kla, that is, the switch of a pro-inflammatory or an anti-inflammatory role of histone Kla in macrophages. As a final note, we also need to trace whether there is a lactate-derived paracrine mechanism between highly glycolytic macrophages and their nearby cells, which might promote their crosstalk during the metabolic switch of macrophages. We therefore look forward to solving these issues and opening up new avenues for mechanistic investigations.

### 5.3 Lactylation and glycolysis

Multiple studies have highlighted the interactions between metabolic regulation and histone modifications (Tan et al., 2011; Sabari et al., 2017). Particularly, some histone acylations, including acetylation, butyrylation, propionylation, and 2-hydroxyisobutyrylation, can be regulated by the changes of glycolysis and OXPHOS(Liberti and Locasale, 2020; Dai et al., 2022). In 2019, Zhang and others first observed a time-dependent increase of histone Kla in a pro-inflammatory M1 macrophage polarization model, highlighting the importance of highly glycolytic activity during histone lactylation (Zhang et al., 2019). Histone lactylation therefore offers new insights into the role of glucose metabolism in their crosstalk with histone modifications. Although an increase of histone lactylation is evident in almost all cases experiencing highly glycolytic activities (such as hypoxia, inhibition of OXPHOS, or feeding more glucose) according to their observations, the mechanistic basis of the interaction between glycolysis and protein lactylation remains to be determined. Consistent to their reports, Li et al. showed that Glis1 facilitated the metabolic remodeling from OXPHOS to glycolysis during the cascade of somatic cell reprogramming. As a result, the accumulated lactate and upregulated histone lactylation (H3K18la) epigenetically modulated the transcription of genes involving cell fate determination (Li et al., 2020) (Figure 2). Similarly, Yang et al. found that hypoxic *in vitro* culture reduced expression of *Ldha* in the mouse blastocysts, along with the attenuated glycolysis, thereby decreasing lactate and histone lactylation levels in pre-implantation embryos (Yang et al., 2021). However, Zhang et al. surprisingly found that glucose metabolism negatively regulated protein lactylations in the protozoan parasite *T. brucei* (Zhang N. et al., 2021). Contrary to the previous studies, they observed a gradual reduction in lactate and lactylation levels of trypanosome along with the increasing glucose concentration and glycolytic rate. They argued that this phenomenon could be attributed to the discrepancy in lactate source among *T. brucei* and other eukaryotes (Zhang N. et al., 2021). Due to the lack of LDH and glyoxalase 1 (GLO1), trypanosome lactate is derived from the glyoxalase pathway, in which MGO is mainly converted to l-lactate rather than d-lactate. Moreover, with the robust substrate saturable uptake activity, trypanosome then experienced a reduction in lactate after the glucose concentration increased, eventually resulting in the downregulation of lactylation.

Additionally, lactylation also serves as a regulator of glycolysis via two branches. On one hand, enzymes involved in glycolysis could be lactylated directly. Gaffney et al. identified 350 lactoyllysine modified proteins in HEK293 cells, most of which were associated with glycolysis, such as HK1, Aldolase A/C, PGK1, ENO1, PKM(Gaffney et al., 2020) (Table 2). Zhang et al., also observed that seven glycolytic enzymes were lactylated on multiple sites in trypanosome, some of which govern their activities (Zhang N. et al., 2021). For example, Aldolase was lactylated at the catalytic site of the enzyme, which might account for its activity changes. Further, the lactylation of PGK and PKM may also contribute to the catalytic process accompanying ATP production. On the other hand, histone lactylations, in turn, can also enrich at promoters of multiple glycolytic genes, where they play a critical role as glycolysis regulators. Pan et al. found that H4K12la was enriched at the promoters of genes associated with glycolysis, including hypoxia inducible factor 1 subunit alpha (*Hif1a*), *Pkm*, and *Ldha* etc. in microglia (Pan et al., 2022) (Figure 2).

Indeed, understandably, histone lactylation has a negative feedback regulatory mechanism toward glycolysis. Gaffney et al. found that when exposed to 50 uM MGO for 6 h, GLO2^−/−^ cells displayed an increase in lactoylation but a relative decrease in glycolytic metabolites downstream of dihydroxy acetone phosphate/3-phosphoglyceraldehyde, indicating a negative feedback mechanism serving to regulate glycolytic flux under hyperglycemic or Warburg-like conditions (Gaffney et al., 2020). Similarly, increased histone lactylation levels in NSCLC cells can also down-regulated the expression of glycolytic genes, such as *HK1*, glucose-6-phosphate dehydrogenase (*G6PD*) and *PKM*, attenuating glycolysis and maintaining mitochondrial homeostasis (Jiang J. et al., 2021).

Certainly, in some pathological conditions, histone lactylation at glycolytic gene promoters can also produce a positive feedback loop for its egoistic self-regulation. Pan et al. found that H4K12la was elevated at the promoters of glycolytic genes, including *Hif1a*, *Pkm*, and *Ldha* etc., which further activated glycolysis and facilitated the formation of vicious feedback loop, termed glycolysis/H4K12la/Pkm2, in inflammatory microglia that drive the pathogenesis of AD (Pan et al., 2022) (Figure 2).

### 5.4 Crosstalk between lactylation and other acylations

PTMs confer new paradigm of bioactivity and functions to proteins and play central roles in myriad biological pathways (Millar et al., 2019). Crosstalk among distinct PTMs have been confirmed in several organisms, meaning that one PTM can facilitate or repress the formation of another or exert their functions in a combinatorial way (Csizmok and Forman-Kay, 2018; Dai Vu et al., 2018; Zhang et al., 2020). Growing evidence indicates that there is a crosstalk between histone lactylation and acetylation (Zhang et al., 2019; Li et al., 2020; Yang et al., 2021; Yang K. et al., 2022; Dai et al., 2022; Moreno-Yruela et al., 2022).

On one hand, both lactylation and acetylation prefer lysine as the residue for PTM, which leads to their competition. The extent of lactylation and acetylation also reflects the level of lactate and acetyl-CoA, the ratio of which has been used for evaluating dominant energy utility in cell metabolism. In 2019, Zhang et al. first observed the different temporal dynamics between histone Kla and Kac during M1 macrophage polarization (Zhang et al., 2019). A slower kinetics of histone Kla (24 h), compared to histone Kac (6 h), were observed, indicating a strong competence of histone Kac and Kac in a physiological condition. Further, data from Chromatin Immunoprecipitation-seq revealed that 68% (1223/1787) of all genes detected in analysis marked by increased H3K18la but not H3K18ac, suggesting an obvious difference in genes enriched by Kla or Kac on a same lysine residue (H3K18) (Zhang et al., 2019). In another study, investigators found an evident distinction of the dynamic distribution between histone Kla and Kac in oocytes and early embryos in mouse (Yang et al., 2021). Particularly, when exposed to hypoxia state, both H3K23la and H3K18la in the blastocysts experienced a significant decrease, compared to the stable H3K23ac and H3K18ac. These decreases subsequently contributed to the robust impairment of the developmental potential of pre-implantation embryos in mice.

On the other hand, lactylation and acetylation share the catalytic ‘writer’, termed p300, in cells, enabling their co-modification on the same or different proteins under both physiological and pathological conditions, which indicates a temporally and spatially way cooperatively controlled by lactylation and acetylation. Consistent with that, Li et al. found histone Kla (H3K18la) and Kac (H3K27ac) cooperatively modulated the expression of pluripotency genes, including *Oct4*, *Sall4* and *Mycn*, during the process of somatic cell reprogramming (Li et al., 2020). Further, Hmgb1, the so-called alarmins proteins in stressed or activated inflammatory cells, can also be co-modified by L-Lactyl-CoA and acetyl-CoA in polymicrobial sepsis (Yang K. et al., 2022). More interestingly, several studies reported that histone Kla can also partly share the catalytic ‘erasers’ of protein acetylation, especially for HDAC1/3 (Jennings et al., 2021; Moreno-Yruela et al., 2022; Zessin et al., 2022; Zu et al., 2022).

In addition to lysine acetylation, many other acylated modifications, such as crotonylation, 2-hydroxyisobutyrylation, succinylation, and malonylation, can also co-modify target proteins concurrently with lysine lactylation, suggesting potential crosstalk among these modifications (Gao et al., 2020; Meng et al., 2021). By the proteome-wide analysis, Meng et al. first found substantial overlaps between lactylation and other acylations in rice (Meng et al., 2021). According to their acylation data sets, 60%, 33%, 29% and 25% of lactylated proteins were co-modified with 2-hydroxyisobutyrylation, succinylation, acetylation, and malonylation, along with 47%, 24%, 8% and 10% of lactylated lysine modified at the identical sites, respectively. Strikingly, they even found 49 proteins were co-modified by five types of acylations, suggesting that many lysine residues can be modified by more than one acyl groups (Meng et al., 2021). Similarly, Gao et al. reported that a total of 143 sites on 83 lactylated proteins in *B. cinerea* were commonly co-modified by lactylation, crotonylation and 2-hydroxyisobutyrylation, furtherly indicating the high conservation of acylated modifications across plant, human, and fungi (Gao et al., 2020). They also observed the mechanical and functional correlations among these paired modifications, highlighting the non-randomly formation of the associated pairs. Intriguingly, these acylations, such as crotonylation, 2-hydroxyisobutyrylation, also share p300 as an indicative writer protein, facilitating the process of crosstalk (Figlia et al., 2020). However, we do not yet know how the writer chooses which PTM mark to write in. One plausible explanation is that the active acetyltransferases can randomly modify lysine residues by acyl groups depending on the availability of the corresponding metabolites, whereas different acylations compete for lysine residues as a mode of negative crosstalk during specific biological modulations. Collectively, additional studies are required to definitively interpret the possible competition, crosstalk and functional interaction among diversified acylations on the same sites of target proteins.

### 5.5 Targeted therapy

Drugs targeting processes of acylated modification have been used clinically and achieved remarkable objective responses, especially in antitumor treatment. The classic example is the deacetylase inhibitors, which is first proposed with the discovery of valproate for the treatment of epilepsy (Ibhazehiebo et al., 2018). Recently, several deacetylase inhibitors, such as Vorinostat (Ogura et al., 2014), Belinostat (O'Connor et al., 2015), Romidepsin (Zinzani et al., 2016)and Panobinostat (Kaufman et al., 2019), have been approved by the US Food and Drug Administration for the treatment of lymphoma and myeloma. Similarly, protein lactylation can facilitate oncogenic signals (Yu et al., 2021; Xiong et al., 2022), also playing an undoubtedly therapeutic targets for tumors.

Lactate has been proposed as a novel oncotherapeutic target (Feng et al., 2018; Pucino et al., 2018; Spencer and Stanton, 2019; Taddei et al., 2020). To date, most drugs target the lactate transporters, such as MCT1 and MCT4, which are undergoing preclinical studies or clinical trials (Marchiq et al., 2015; Feichtinger and Lang, 2019). Histone lactylation, however, was recently defined as an epigenetic mark of the glycolytic switch, which was sensitive to exogenous and endogenous lactate levels, suggesting novel therapeutic opportunities for tumors (Dai et al., 2022). Yu et al. revealed that histone lactylation accelerated tumorigenesis through activating m^6^A reader protein, YTHDF2, indicating a potential target of histone lactylation for treating ocular melanoma (Yu et al., 2021). Subsequently, Xiong et al. found that lactate in tumor microenvironment promoted the sustained immunosuppressive activity of tumor-infiltrating myeloid cells via the lactylation-Mettl3-m^6^A-Jak/Stat3 path, thus providing new clues for the development of immunotherapeutic strategies in colorectal cancer (Xiong et al., 2022).

In addition to neoplastic disease, histone lactylation also provides therapeutic options for inflammatory disease. Recently, Pan et al. revealed that PKM2 inhibitors (shikonin or compound 3K) targeting the glycolysis/H4K12la/Pkm2 loop reduced the extent of reactive microgliosis, partially reversed the amoeboid morphology of microglia, and significantly improved spatial learning and memory in AD models, suggesting a promising strategy for mitigating neuroinflammation in AD and other neurodegenerative diseases (Pan et al., 2022). Alternatively, one study revealed the key role of Kla in modulating fungal virulence, indicating that the in-depth exploration of functions in Kla may help to prevent and control gray mold disease (Gao et al., 2020). A recent study also proposed that lactate sensor GPR81-mediated signaling decreased circulating exosomal Hmgb1 levels, highlighting a promising therapeutic target of lactate-associated signaling in polymicrobial sepsis (Yang K. et al., 2022).

To conclude, the discovery of lactylation explores new biological and functional considerations, paving the way for studying the underlying mechanisms of the diverse roles of lactate. Intensive investigation of the comprehensive regulatory mechanism of lactylation is beneficial to the development of therapeutic strategies.

## 6 Conclusion and perspectives

Protein lactylation, a novel PTM, is a new contributor to the epigenetic landscape and governs diversely physiologic and pathologic settings through comprehensive mechanisms. Recent 3 years, pivotal roles of protein lactylation in modulating the process of complex biological functions, including inflammation, fibrosis, neoplastic disease, AD, stemness maintenance, embryonic development, and neuromodulation have been revealed. Research on lactylation, however, is being in its infancy, with some critical questions remained.

Up to now, controversies in ‘lactylgenesis’ machinery, the key mechanism governing the switch of a pro-inflammatory or an anti-inflammatory role of histone Kla in macrophages, and crosstalk between lactylation and many other biological processes (such as glucose metabolism and multiple modifications in RNA, DNA or protein) are largely unsolved. Further, identification of protein lactylation sites in cross-species, especially in human and mouse, could refine the Species-Wide Lactylation Atlas, helping us to figure out more functional and mechanistic pointbreaks in future lactylation research. Future studies creating knock-in point mutants of lysine lactylation sites that render these proteins incapable of lactylation may help to fully elucidate their multiple important functions. Last but not least, elevated lactate levels have been observed in many organs (e.g., liver, muscle) and in a variety of conditions (e.g., exercise, lactic acidosis). Whether progressive and transient accumulation of lactate leads to profound alterations in tissue/organ phenotype and function via protein lactylation remains unknown. Additional research describing protein lactylation across tissues/organs within an intact animal undoubtedly will be of great interest to this field. In general, exploring the functional phenotype and mechanisms of protein lactylations in depth will further broaden our horizons in lactate metabolism and epigenomics and boost the generation of new strategy for disease treatment.
